# Wildlife-friendly farming increases crop yield: evidence for ecological intensification

**DOI:** 10.1098/rspb.2015.1740

**Published:** 2015-10-07

**Authors:** Richard F. Pywell, Matthew S. Heard, Ben A. Woodcock, Shelley Hinsley, Lucy Ridding, Marek Nowakowski, James M. Bullock

**Affiliations:** 1 NERC Centre for Ecology and Hydrology, Wallingford OX10 8BB, UK; 2 Wildlife Farming Company, Bicester OX26 1UN, UK

**Keywords:** sustainable intensification of agriculture, ecosystem services, agri-environment schemes, pest control, pollination

## Abstract

Ecological intensification has been promoted as a means to achieve environmentally sustainable increases in crop yields by enhancing ecosystem functions that regulate and support production. There is, however, little direct evidence of yield benefits from ecological intensification on commercial farms growing globally important foodstuffs (grains, oilseeds and pulses). We replicated two treatments removing 3 or 8% of land at the field edge from production to create wildlife habitat in 50–60 ha patches over a 900 ha commercial arable farm in central England, and compared these to a business as usual control (no land removed). In the control fields, crop yields were reduced by as much as 38% at the field edge. Habitat creation in these lower yielding areas led to increased yield in the cropped areas of the fields, and this positive effect became more pronounced over 6 years. As a consequence, yields at the field scale were maintained—and, indeed, enhanced for some crops—despite the loss of cropland for habitat creation. These results suggested that over a 5-year crop rotation, there would be no adverse impact on overall yield in terms of monetary value or nutritional energy. This study provides a clear demonstration that wildlife-friendly management which supports ecosystem services is compatible with, and can even increase, crop yields.

## 1. Introduction

Rapid human population growth and changes in diet preferences are driving a rising and unsustainable demand for food globally [1]. This, coupled with apparent yield plateaus for many major crops [2], has led to concerns about expansion of agricultural land resulting in the loss of semi-natural habitats. This process is also likely to lead to significant intensification of agricultural practices to the detriment of both the environment and biodiversity, including many ecosystem services that support human well-being [3]. Recent commentaries suggest that ecological intensification of agriculture might offer a solution to this pressing challenge [4,5]. This concept is based on devising practical management strategies that integrate and enhance the ecosystem functions associated with crop production, such as pollination and pest control, into commercial farming systems without detriment to other services or natural capital. However, there is a dearth of knowledge about how one might implement such management in practice, or whether it will enhance crop production. This knowledge gap means that ecological intensification remains a largely theoretical concept.

By contrast, there is compelling evidence that wildlife-friendly farming practices, aimed at reducing the negative impacts of intensive agriculture by implementing conservation actions in farmed landscapes, can be effective in conserving and restoring biodiversity [6–8]. In particular, habitat management practices, both in- and off-field, can support taxa that potentially provide services which enhance food production, such as native pollinators and predators of crop pests [9,10]. Often these practices are applied with specific conservation targets in mind, for example, to increase the abundance of farmland birds, and have limited focus on delivering ecosystem services. While there is evidence that enhancing native biodiversity in this way could play a role in increasing agricultural productivity [10–13], other studies show that it does not always lead to improved ecosystem service delivery [14]. Some studies have linked crop yield benefits to the proximity of existing pristine habitats [15,16], and a few have linked creation of wildlife habitat to increased yield in fruit crops [17,18].

To be effective, ecological intensification of agricultural systems will require the development of packages of management prescriptions that work synergistically to increase production, for example, by both providing nesting and foraging habitat for key crop pollinators and enhancing soil organic matter. At the same time, it is important that these packages do not excessively constrain crop management or compromise delivery of other ecosystem services. Their effective implementation requires clear demonstration of benefits, together with information and advice to encourage widespread practitioner uptake [19]. In particular, yield benefits of any ecological intensification actions should be evaluated against potential costs to the farmer, such as those resulting in land lost from production, and the effort required to create and maintain good quality habitats.

We undertook a 6 year farm-scale randomized block experiment to test whether the removal of small amounts of land from food production for the creation of wildlife habitat increased the yield of globally important food crops (grains, oilseeds and pulses) compared with a business as usual (BAU) control. Critically, we asked whether the enhanced yield is sufficient to compensate for the cropping area lost to habitat creation, and thus provided some of the first evidence for commercially viable ecological intensification.

## 2. Material and methods

### (a) Study site

The experiment was conducted on the 900 ha Hillesden Estate in Buckinghamshire, central England (51.95° N, 01.00° W). The farm was situated on heavy clay soils with a relatively flat topography and was characterized by large (10–20 ha), homogeneous arable fields cropped under a simple rotation of autumn-sown first wheat (*Triticum aestivum* L. (Poaceae)) followed by break crops of either oilseed rape (*Brassica napus* L. (Brassicaceae)) or field beans (*Vicia faba* L. (Fabaceae)) then back into wheat. Second wheat crops were rarely sown (5% of fields). In most years, approximately 50% of the cropped area was wheat and 50% break crop (typically 13% beans; 37% oilseed rape). All crops were managed consistently across the farm regardless of experimental treatment with conventional inputs of fertilizers and pesticides which aimed to maximize yield. Typical crop agronomy is represented in the electronic supplementary material though there were small variations between years to account for factors such as weather and pest outbreaks.

### (b) Experimental design

Between September 2005 and 2011, a randomized block experiment was implemented to examine the effects of converting differing proportions of arable land to wildlife habitat to support declining farmland biodiversity according to the rules of the English agri-environment schemes. Some of the prescribed habitats are also known to benefit species associated with crop production, particularly pollinators and natural enemies of crop pests. Two wildlife enhancement treatments were compared to a continuation of intensive conventional agriculture in which no land was removed from production as a ‘BAU’ control. The farm was divided into five contiguous replicate blocks of 150–180 ha depending on field size. Each of the three treatments was applied at random within each block to discrete groups of fields, with each group having a combined area of 50–60 ha. In the BAU control, fields were cropped to the edge. The first comparator was the ‘ELS' treatment, simple habitat enhancement based on the Entry Level Stewardship (ELS) agri-environment scheme [20] which represented typical practice of many farmers in this region. Under ELS, 3% of the usable cropped land was removed from production (equivalent to approx. 1% of the whole area) to create wildlife habitats at field edges and in awkward field corners, comprising: (i) 1.2 ha of 6 m wide field margins sown with four tall grass species, which provide overwintering sites for invertebrate predators [21] and bumblebees [22], and (ii) a single patch of 0.3 ha sown with short-lived plants designed primarily to supply winter seed resources for farmland birds, but also known to provide early season floral resources for crop pollinators [23] (see the electronic supplementary material, table S1 for full details of seed mixtures). The second comparator treatment was ELS extra (‘ELSX’), which included other wildlife habitats in addition to those in ELS and had a greater proportion (8%) of cropped land out of production (equivalent to approx. 5% of the total area). This comprised three discrete 0.5 ha patches sown with a diverse mix of perennial native wildflowers and fine-leaved grasses (29 species) to provide good quality foraging, nesting and refuge habitat for pollinators and natural enemies of crop pests [24,25]. A further three 0.5 ha patches were sown with different mixes of short-lived plants for birds and pollinators. The remaining converted area (1.4 ha) comprised margins sown with either four legume species designed to provide mid- to late-season floral resources for pollinators [25], or with a mix of five tall grasses and six nectar-providing forb species.

### (c) Crop yields

**Figure 1.**
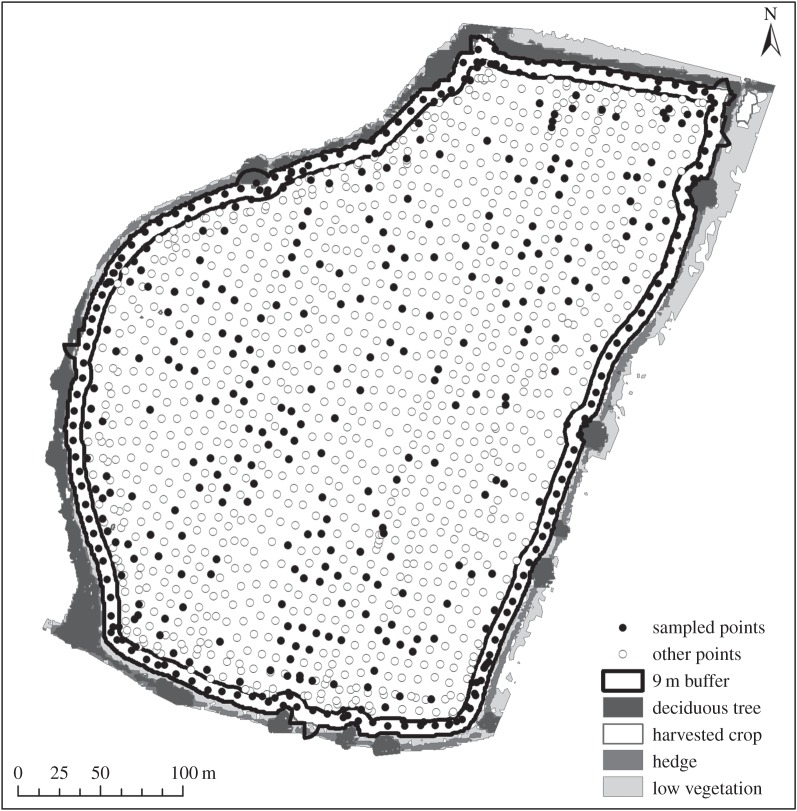
A detailed crop yield map used to compare yield at the edge (0–9 m) with the rest of the field.

Yield of the three crop types was measured in each field (*n* = 56) in each year 2006–2011 using the on-board yield meter (Quantimeter) on the CLAAS Lexion 580 + combine harvester (CLAAS KGaA mbH, Harsewinkel, Germany). The yield meter measures clean grain volume in the elevator to calculate the yield values. Field tests have shown this system is more than 97% accurate [26]. Between 2007 and 2011, individual yield measurements were geo-referenced using a ground station corrected GPS signal (accuracy ± 15–30 cm). This produced a point-based yield map for each field (figure 1). These detailed data were used to quantify differences in crop yield between the field edge and corners, and the rest of the field. A buffer equivalent to a single combine cutter bar width (9 m) was drawn around the edge of all 17 fields that were cropped to the edge in the control treatment. The number of yield points falling inside this buffer was calculated for each field in each year (85 field × year combinations). These were compared to approximately the same number of yield values selected at random from the rest of the field. This spatially explicit sampling of crop yields enabled an accurate estimate of the potential yield loss from wildlife habitat creation at the edge of the field.

Yield values were summed to produce crop yield data (tonnes) for each field in each year. Crop yields were divided by: (i) the cropped area (ha) of the field, i.e. the area of the field minus the area removed for wildlife habitat creation and (ii) the whole field area (ha). The cropped area yield (i) indicates whether the crop yield per unit area was altered by the presence of wildlife habitat. The whole field yield (ii) would indicate whether using part of the field for wildlife habitat rather than for cropping decreased the overall yield from the field. These yield values were divided by the regional average yield per hectare for winter wheat and oilseed rape in the relevant year based on statistics from the National June Survey of Agriculture and Horticulture and published by the UK Department for Environment, Food and Rural Affairs (Defra) [27]. In the case of field bean, we used national average yield data, as regional data were not available. The resulting ratios were used in the analyses rather than yield per hectare data, because they set the study yields in the context of what might be expected in the region in terms of a yield ‘deficit’ or ‘surplus'. Furthermore, in these analyses we focus on the treatment main effects and interactions, and the use of these ratios reduces the variation due to main effects of crop type (e.g. wheat is much higher yielding than the broadleaf crops) and year.

### (d) Crop pollinators and pest natural enemies

Between 2007 and 2010, the abundance and diversity of bee species was recorded along 55 transects (each 50 × 2 m) situated in habitats typically found in each of the three treatments. These were recorded in May and June to be coincident with crop flowering. In the BAU treatment, transect counts were made along the crop edge. In ELS, transects were recorded at the crop edge, along grass margins and in the wild bird seed mixture. In ELSX, counts were in the crop, along nectar flower margins and patches sown with wildflowers, and a variety of wild bird seed mixtures. Abundances and species richness were averaged across the different sampled habitats in each treatment to provide a comparable measure of the pollinators in BAU, ELS and ELSX treatments. Counts were carried out between 10.00 and 17.00 when weather conditions conformed to the Butterfly Monitoring Scheme (BMS) rules (temperature above 13°C with at least 60% clear sky, or 17°C in any sky conditions) [25]. Honeybees were counted and bumblebees were recorded to species level. Both groups are considered important pollinators of oilseed rape and field beans [28].

Ground beetles were sampled for two four-week periods in May and July 2008 using pitfall traps. A field was selected at random within each treatment and six pitfall traps, each separated by 2 m, were placed 3 m into the crop from the edge. Individual traps (diameter 75 mm × depth 105 mm) were filled with a 50% solution of ethylene glycol with a small volume of detergent. Traps were emptied at two-week intervals and catches summed across all traps within a site for both sampling periods. Ground beetles were identified to species level and classified as either predominantly predatory or phytophagous [29].

### (e) Statistical analysis

Although the experiment had a randomized block design with treatments assigned permanently to groups of fields within five blocks, the yearly rotation of the three crop types (wheat, oilseed rape and beans) was done independently for each field, in accordance with the farm business requirements. Thus, the crop rotation unbalanced the design and the grouping of fields along with measurements over consecutive years meant data were not independent. We addressed these issues by using linear mixed models (Proc Mixed in SAS v. 9.3), with block, field and year as random effects. Field was nested within block, and year was nested within field; the latter was preferred to a repeated measures approach as the crop rotation meant that year was confounded with crop type. Fixed effects included in the cropped area ratio and the whole field ratio models were treatment (BAU, ELS, ELSX), crop type and treatment × crop and treatment × year interactions. These models were fitted using restricted maximum likelihood (REML), which was used to test for the significance of each fixed effect. Least-square means and associated standard errors for the fixed effects were calculated to accommodate the unbalanced design, as they are estimates of the marginal means over a balanced population. Post hoc pairwise differences between least-square means were calculated using two-tailed *t*-tests. Examination of residuals confirmed that the data were normally distributed.

We also used linear mixed models to examine differences in the abundance and species richness of crop pollinators and pest control species between treatments. For pollinators, block and year were included as random effects, with treatment and treatment × year interactions as fixed effects. The model for pest control species did not include a year term. Model-based estimation of fixed effects was performed using REML. Simplification of models was undertaken using deletion of least significant effects from a saturated model. Poisson distribution and log link functions were used to assess abundance and species richness responses for the ground beetles. Pollinator abundances and species richness were based on averaged values across different margin and crop types within each BAU, ELS and ELSX treatment and were found to be normally distributed.

## 3. Results

**Figure 2.**
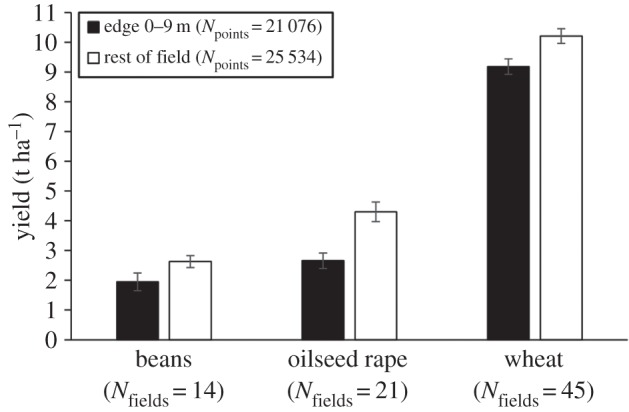
Crop yield (mean ± s.e.) measured at the edge of the field (0–9 m) and the rest of the field for beans, oilseed rape and wheat between 2007 and 2011 for the 17 fields in the BAU control. *N*_fields_ = the number of fields a given crop occurred in the BAU treatment between 2007 and 2011; *N*_points_ = the total number of points used to calculate mean yields.

All three crops showed consistent and marked reductions in yield at the field edge (0–9 m) compared with the rest of the field in the BAU treatment (figure 2). Yield of winter wheat was reduced by a mean of 10.1% (±1.1%), beans by 25.9% (±7.5%) and oilseed rape by 38.2% (±3.8%).

**Figure 3.**
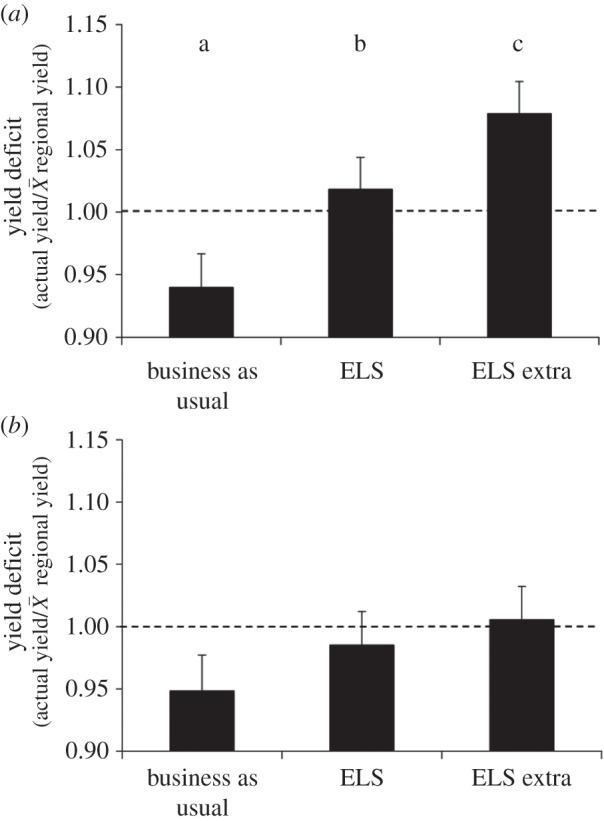
Crop yield (mean ± s.e.) as a ratio of regional and national yields averaged for all crops (wheat, oilseed rape and field beans) and all years (2006–2011) for (*a*) cropped area and (*b*) whole field net of land removed for wildlife habitat creation. Treatments with a different letter are significantly different (*p* < 0.05).

Over the 6 years, the average yield for all crops (wheat, oilseed rape and beans) in the cropped area was significantly enhanced by creation of wildlife habitats on 3% of land (ELS) and was further increased by creation of habitat on 8% of land (ELSX) (*F*_2,68.3_ = 7.10; *p* < 0.01; figure 3*a*). However, there were no differences between the three treatments in terms of the whole field ratio (i.e. including the land removed for habitat creation) (*F*_2,68_ = 1.08; *p* > 0.05; figure 3*b*), which suggests that the removal of up to 8% of land from production resulted in no net loss of yield at the field level, as *per* unit area productivity was increased.

**Figure 4.**
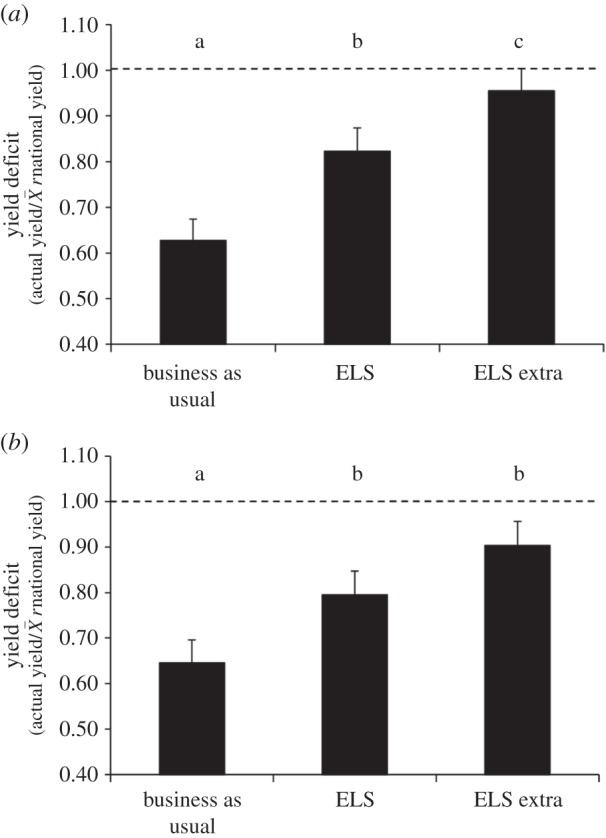
Yield of field beans (mean ± s.e.) as a ratio of national yields averaged over all years (2006–2011) for (*a*) cropped area and (*b*) whole field net of land removed for wildlife habitat creation. Treatments with a different letter are significantly different (*p* < 0.05).

The crop types differed in their responses to the treatments for both the cropped area (crop × treatment *F*_4,251_ = 4.16; *p* < 0.01) and the whole field (*F*_4,252_ = 4.79; *p* < 0.001) ratios. Post hoc comparison of least-square means revealed no overall difference (at *p* > 0.05) between the treatments for winter wheat or oilseed rape (see the electronic supplementary material, figures S1 and S2), which demonstrates no yield loss from habitat creation in these crops. However, there was a strong treatment effect for beans, with yield in the cropped area significantly higher in the ELS compared with BAU, and in ELSX compared with either ELS or BAU (figure 4*a*). Yield for the whole field was significantly greater in both ELS and ELSX compared with BAU (figure 4*b*). Thus, for beans, fields with wildlife-friendly habitats had higher overall yields than fields of the same size with no habitats; this yield increase was 25% and 35%, respectively, for ELS and ELSX relative to BAU.

**Figure 5.**
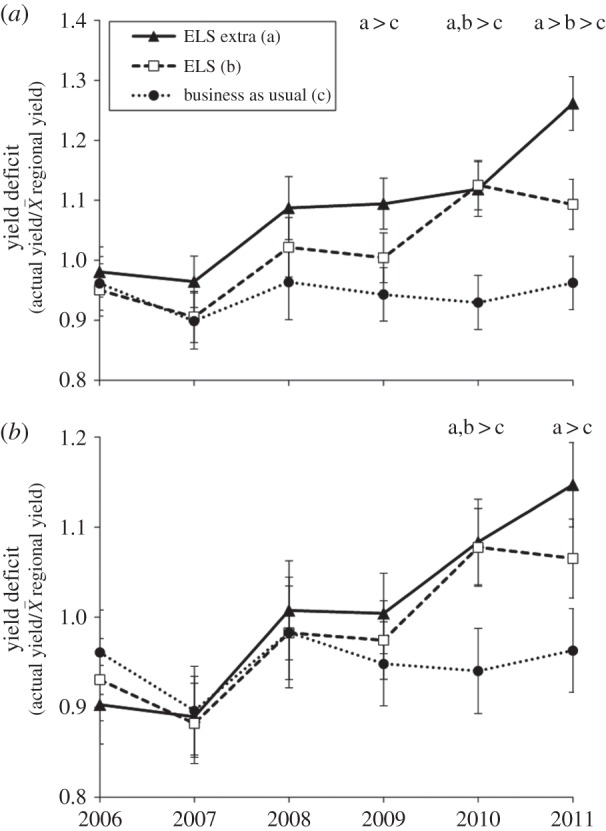
Trends in crop yield (mean ± s.e.) as a ratio of regional and national yields averaged for all crops (wheat, oilseed rape and field beans) for (*a*) cropped area and (*b*) whole field net of land removed for wildlife habitat creation. Treatments with a different letter are significantly different (*p* < 0.05).

The treatment effects also increased over time. For the cropped area ratio, there were no treatment differences for the first 3 years of the experiment. After 4 years, treatment effects became manifest and these became larger over the following years in the order ELSX > ELS > BAU (year × treatment *F*_15,234_ = 4.46; *p* < 0.001; figure 5*a*). For yield over the whole field, treatment differences developed more slowly, but followed similar patterns (*F*_15,235_ = 3.50; *p* < 0.001; figure 5*b*).

The individual crops had different monetary and energy values and so an analysis of the consequences for the farmer of having wildlife margins required translating tonnes per hectare for each crop into common units, which we did in terms of nutritional energy content, or their monetary value. Mean nutritional energy values (MJ/t) for each of the three crops were taken from compilations of published information [30]. Similarly, the monetary value (€/t) of each was calculated using published Gross Margin data [31]. This allowed us to consider treatment effects on combined yield across all crops, which we did in terms of a standard rotation of 3 years wheat, 1 year oilseed rape and 1 year beans (in any order). We took the least-square mean whole field yield estimates and standard errors for each crop in each treatment and multiplied these by mean energy or monetary values for that crop. We then calculated the weighted average yearly energy or monetary yield over the 5-year rotation. This showed little difference in either measure of overall yield among the three treatments (see the electronic supplementary material, figure S3*a*,*b* and table S2), with the lower energy and monetary yield for wheat in the ELS and ELSX treatments being balanced by the higher yields of beans compared to BAU.

**Figure 6.**
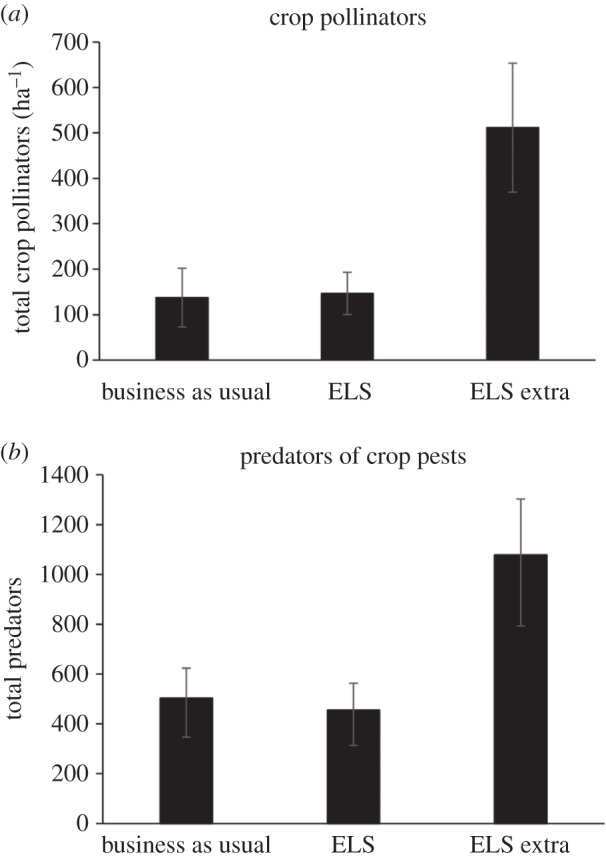
Mean (±s.e.) abundance of (*a*) oilseed rape and field bean pollinators (*Apis mellifera* and *Bombus* sp.) per hectare in each treatment between 2007 and 2010 and (*b*) predatory Carabid beetles recorded in each treatment in 2008.

Overall there were significant differences in abundance (*F*_2,8_ = 9.48; *p* < 0.01; figure 6*a*) and species richness (*F*_2,8_ = 5.33; *p* < 0.05) of crop pollinators among treatments. Abundance and richness were higher in ELSX compared with either ELS or BAU. There were no differences between ELS or BAU. Similarly, the abundance of predatory ground beetles was significantly higher in ELSX than the other treatments (figure 6*b*) (*F*_2,3_ = 309.6, *p* < 0.001), although there were no treatment effects on their species richness (*F*_2,3_ = 0.77, *p* > 0.05).

## 4. Discussion

This study has demonstrated no significant loss of yield per hectare for globally important arable crops when up to 8% of cropped land is removed from production for the creation of wildlife habitat. Critically, wildlife-friendly farming appeared to increase the yield of an important protein crop (field bean), proving the concept of ecological intensification of agriculture is achievable on a large commercial arable farm in northwest Europe. This finding complements recent studies in fruit crops in South Africa and North America showing that production can be increased by planting native flowers [17,18]. Together these findings have important implications for future sustainable intensification of agriculture, and supports the argument that lower yielding and otherwise compromised areas of fields can be better used as non-crop habitats to provide services supporting crop production, benefits for farmland biodiversity, and the protection of water and soil resources (known as land sharing).

These beneficial effects on yield might be explained by a combination of ceasing to plant on land with the most severe constraints on crop growth, and the spill-over of beneficial agro-ecological processes from adjacent wildlife habitats. The yield reduction of wheat at the field edge in our study (10%) is comparable with that of earlier studies (7%) [32], although the more extreme reductions in yield of oilseed rape and beans have not been reported previously. Yield reduction at the field edge may be due to a number of interacting factors, including soil compaction, competition for light and water resources with adjacent hedges and trees, and increased pressure from pests and weed species [32].

The higher abundance and diversity of crop pollinators supported by wildlife-friendly farming practices provides correlative evidence to explain the increased yields of insect-pollinated field crops. Moreover, recent studies at Hillesden of bumblebee populations using molecular markers have shown that nest densities are higher and average foraging distances are lower within the ELSX treatment, which has a high proportion of bee-friendly habitat, than areas where this habitat is absent [33]. Commercial bean crops comprise a mixture of self-fertile hybrid plants and inbred plants that must be cross-pollinated to set seed. Exclusion and containment experiments suggest that yield of this crop is increased by 30% by insect pollinators [34], and that long-tongued bumblebees (*Bombus* sp.) are more effective pollinators than short-tongued bumblebees and honeybees (*Apis mellifera* L.) [35]. Insect pollination has also been shown to be a key factor limiting the yield of this crop in large commercial fields typical of our study farm [36]. A high proportion of the habitats created under the ELSX treatment (wildflower corners, pollen and nectar margins, etc.) are known to particularly benefit long-tongued bumblebees [24]. By contrast, wildlife-friendly farming practices appeared to have no beneficial effects on oilseed rape, despite bagging studies showing that insect pollination can boost the yield and crop quality [37]. Modern rape varieties are fully fertile and self-pollinating to a high degree, making pollination effects relatively small and therefore difficult to detect, especially over such a large-scale experiment. Moreover, few of the habitats created in this study provide the early season pollen and nectar habitats that might directly benefit oilseed rape pollinators, although many provide important overwintering and nesting habitat [22].

The tall grass field margins and floristically enhanced field corners created in this study are likely to attract and support a large number of both flying and epigeal natural enemies of economically damaging crop pests, such as cereal grain aphids (*Sitobion avenae* L.) [24,38]. Indeed, the results of limited pitfall trapping confirmed significant increases in ground beetles in the ELSX treatment. These will feed on the ground-dwelling larvae and overwintering adults of pea and bean weevil (*Sitona lineatus* L.), a major pest of field bean, reducing the population by as much as 30% [39]. However, there were no detectable benefits or dis-benefits of wildlife-friendly farming on the yield of the dominant wind-pollinated crop, winter wheat. This lack of a positive effect on cereal yield might be explained by the large field size in this study (mean 13.1 ± 0.8 ha) and the rapid fall-off of bio-control from epigeal predators away from the field edge [40]. This might be overcome by creating in-field grass banks (‘beetle banks') as a means of encouraging beneficial predatory insects into the field centre [41]. While not measured in this study, other research has shown that flying predators supported by field margins were as effective as all predators combined in controlling grain aphid and reduced numbers by 90%, whereas epigeal predators only achieved a reduction of between 40 and 18% [42]. Flying predators will be more mobile and are capable of moving between fields, making the detection of field-scale benefits of wildlife-friendly farming difficult.

By combining the treatment effects on individual crops, we were able to show that creation of wildlife habitat resulted in no loss to the farmer in terms of the monetary or nutritional energy yield across a typical 5-year arable crop rotation. Slightly lower yields of wheat and oilseed rape, owing to planting of habitats on cropland, were counterbalanced by the increased yield (t ha^−1^) of beans by 25% and 35%, respectively, for ELS and ELSX relative to BAU. These apparent benefits to yield and profitability need to be carefully balanced against the cost and practical difficulties in establishing and maintaining this range of wildlife habitats on a commercial farm. There are often conflicts between crop and wildlife habitat management caused by constraints of time and cost of manpower. In the European Union context, agri-environment schemes pay farmers to manage their land for the benefit of particular habitats and species (mean annual expenditure 2007–2013 of €3.33 billion [43]). While we would argue that these payments are important to incentivize farmers to create good quality wildlife habitat, further monitoring and detailed research into the true costs and benefits of this ecological intensification across a range of farming systems and locations could inform future reviews of these support payments to farmers. Any such analysis must also take careful account of the additional indirect benefits of wildlife habitat creation on factors such as water quality, greenhouse gas capture and aesthetic and recreational value of intensively farmed landscapes [3].

Finally, it took around 4 years for the beneficial effects on crop yield to manifest themselves and these appeared to strengthen with time. This could be considered further indirect evidence of biodiversity-mediated benefits to crop production, reflecting the time taken for populations of pollinators and other beneficial insects to respond to wildlife-friendly farming. Recent studies show increases in the numbers of pollinating insects over similar time periods in response to creation of pollen and nectar habitats across a landscape gradient [8]. It would be interesting to measure whether these effects on yield continue to increase with time. Similarly, further research is required to determine more accurately the optimum amount and combination of habitat required at the farm- and landscape-scale to increase yield yet leave sufficient land for food production.

Agricultural productivity has to increase in order to meet the growing demand for food, but this must not be at the expense of biodiversity and ecosystem services associated with human well-being [44]. Our study has demonstrated that it is feasible to remove up to 8% of land from production on a large, intensively managed commercial farm to create a range of beneficial wildlife habitat and maintain yields of key arable crops critically important to food supply in northwest Europe. Indeed our results indicate that yield and profitability of some insect-pollinated crops may even be increased by this approach. However, the policy implications of these findings can only be fully recognized by testing the robustness and generality of such ecological intensification across a wide range of farming systems and situations. There is also considerable scope for the development of improved habitats for ecological intensification based on better understanding of the underlying processes. Better engagement and training of farmers will also be essential for the delivery of these more demanding and complex wildlife habitats [19]. Indeed, recent research suggests that training of farmers is highly effective in improving the quality of wildlife habitat delivered on a farm [45] and this may translate to greater benefits to crop yield.

## Supplementary Material

Additional details of crop angronomy, wildlife seed mixtures, additional analysis of wheat and oilseed rape, analysis of nutritional value and profitability

## Data accessibility

See the electronic supplementary material.
