# Supplementary material for: Wildlife-friendly farming increases crop yield: evidence for ecological intensification
Source: Proc Biol Sci. 2015 Oct 7;282(1816):20151740. doi: 10.1098/rspb.2015.1740 (PMC4614778; doi:10.1098/rspb.2015.1740)
Supplement: Additional details of crop angronomy, wildlife seed mixtures, additional analysis of wheat and oilseed rape, analysis of nutritional value and profitability [file rspb20151740supp1.docx]

**Electronic Supplementary Material**

1. **Full details of crop agronomy**
2. Winter wheat (varieties: Alchemy, Claire, Diego, Duxford, Einstein, Humber, Oakley, Robigus, Santiago, Viscount)
3. Tillage: years 1&2 – non-inversion and ring roll; years 3-5 30% - non-inversion and ring roll, 70% - inversion and ring roll.
4. Drilling Dates: 20^th^ September to 20^th^ October.
5. Pre-emergence herbicides:

0.3 l ha^-1^  flufenacet (400 g l^-1^ a.i) + diflufenican (100 g l^-1^ a.i)

2.0 l ha^-1^  flufenacet (60 g l^-1^ a.i) + pendimethalin (300 g l^-1^ a.i)

2.0 l ha^-1^ prosulfocarb (800 g l^-1^ a.i)

1.5 l ha^-1^ glyphosate (360 g l^-1^ a.i) applied pre-drilling

1. Post-emergence herbicides:

Autumn: 400 g ha^-1^ mesosulfuron-methyl (30g kg^-1^ a.i.) + iodosulfuron-methyl-sodium (6g kg^-1^ a.i.) + mefenpyr-diethyl (90g kg^-1^ a.i.)

Spring: 0.5 l ha^-1^ fluroxypyr (333 g l^-1^ a.i.)

5. Insecticides:

Seed treatment: 0.2 l 100 kg^-1^ of seed prothioconazole (50 g l^-1^ a.i.) and clothianidin (250 g l^-1^ a.i.)

Mollusicide 7 kg ha^-1^ methaldehyde (3%w/w)

0.2 l ha^-1^ alpha-cypermethrin (100 g l^-1^ a.i.)

1. Fungicides:

T0 – 1 l ha^-1^ chlorothalonil (375 g l^-1^ a.i.) +

T0 – 1 l ha^-1^ propiconazole (62.5 g l^-1^ a.i.) +

T0 – 1 l ha^-1^ cyproconazole 50 g l^-1^ a.i.)

T1 – 1 l ha^-1^ boscalid (233 g l^-1^ a.i.) and epoxiconazole (67 g l^-1^ a.i.) +

T1 – 1 l ha^-1^ chlorothalonil (500 g l^-1^ a.i.)

T2 – 0.66 l ha^-1^ epoxiconazole (125 g l^-1^ a.i.) +

T2 –0.3 l ha^-1^ pyraclostrobin (200 g l^-1^ a.i.) +

T2 – 1 l ha^-1^ chlorothalonil (500 g l^-1^ a.i.)

T2 – 0.6 l ha^-1^ prothioconazole (125 g l^-1^ a.i.) and tebuconazole (125 g l^-1^ a.i.)  - *omitted in dry years.*

In years 4&5:  T2 – 1 l ha^-1^ bixafen (75 g l^-1^ a.i.) and prothioconazole (160 g l^-1^ a.i.); T3 – 0.66 l ha^-1^ prothioconazole (275 g l^-1^ a.i.)

1. Fertilisers:

Typically total of 210 kg ha^-1^ N -  in three separate applications

1^st^ application with addition of sulphur (5%) and 80 kg ha^-1^ P +  80 kg ha^-1^ K depending on soil analysis.

B. Winter oilseed rape (varieties: Astrid, Castille, Cubic, DK Cabernet, Expert, Krypton, Lioness, Rascal, Sesame)

1. Tillage: non-inversion and ring roll.
2. Drilling Dates: 20^th^ August to 10^th^ September.
3. Pre-emergence herbicides (not applied every year):

2.0 l ha^-1^  quinmerac (100 g l^-1^ a.i.) and metazachlor (400 g l^-1^ a.i.)

1. Post-emergence herbicides:

2.1 l ha^-1^ propyzamide (400 g l^-1^ a.i.) +

1 l ha^-1^ tepraloxydim (50 g l^-1^ a.i.) OR cycloxydim (200 g l^-1^ a.i.)

When no pre-emergence herbicide applied:

0.3 l ha^-1^ clopyralid (267 g l^-1^ a.i.) and picloram (67 g l^-1^ a.i.)

To control volunteer cereals as required:

0.66 l ha^-1^ propaquizafop (100 g l^-1^ a.i.)

5. Insecticides:

Seed treatment: 2 l 100 kg^-1^ of seed imidacloprid (100 g l^-1^ a.i.) and beta-cyfluthrin 100 g l^-1^ a.i.)

Mollusicide 7 kg ha^-1^ methaldehyde (3%w/w)

0.25 l ha^-1^ alpha-cypermethrin (100 g l^-1^ a.i.)

1. Fungicides and growth regulators:

Late autumn – 0.5 l ha^-1^ difenoconazole (250 g l^-1^ a.i.) or 0.8 l ha^-1^ flusilazole (250 g l^-1^ a.i.) and carbendazim (125 g l^-1^ a.i.)

Spring (mid-flower ) 0.5 l ha^-1^ prothioconazole (275 g l^-1^ a.i.) + 0.66 l ha^-1^ azoxystrobin (250 g l^-1^ a.i.)

Occasionally 1 l ha^-1^ tebuconazole (250 g l^-1^ a.i.) used as Growth Regulator in Spring

1. Fertiliser.

Typically total of 210 kg ha^-1^ N -  in two separate applications

1^st^ application with addition of 80 kg ha^-1^ P +  80 kg ha^-1^ K  depending on soil analysis.

1. Winter beans (variety: Wizzard)
2. Tillage: non-inversion and ring roll.
3. Drilling Dates: 15^th^ October to 15^th^ November.
4. Pre-emergence herbicides (not applied every year):

2.1 l ha-1 propyzamide (400 g l-1 a.i.) +

2.0 l ha^-1^ prosulfocarb (800 g l^-1^ a.i)

1. Post-emergence herbicides:

1.0 l ha-1 glyphosate (360 g l-1 a.i) as a crop desiccant OR

1.5 l ha^-1^ Diquat (240 g l^-1^ a.i.) as a crop desiccant

5. Insecticides:

Mollusicide 7 kg ha^-1^ methaldehyde (3%w/w)

0.25 l ha^-1^ alpha-cypermethrin (100 g l^-1^ a.i.) for Pea and Bean weevil control if required

1. Fungicides:

1.5 l ha^-1^ azoxystrobin. (250 g l^-1^ a.i.) sometimes with

0.75 l ha^-1^ cyproconazole (40 g l^-1^ a.i.) chlorothalonil. (375 g l^-1^ a.i.)

Both repeated three weeks later

1. Fertiliser.

Typically none

Occasionally 40 kg ha^-1^ P + 40 kg ha^-1^ K depending on soil analysis.

Table S1: Full details of the seed mixtures used to create the wildlife habitats

| **Treatment** | **Wildlife habitat** | **Latin name** | **English Name** | **Seed rate**  **(kg ha^-1^)** | **% composition**  **of seed mix** |
| --- | --- | --- | --- | --- | --- |
| ELS | Tall grass | *Dactylis glomerata* | Cock's-foot (var. Prairal) | 6.0 | 30.0 |
| ELS | Tall grass | *Festuca arundinacea* | Tall Fescue (var. Starlett) | 3.0 | 15.0 |
| ELS | Tall grass | *Festuca pratensis* | Meadow Fescue (var. Lifara) | 5.0 | 25.0 |
| ELS | Tall grass | *Festuca rubra* | Slender Creeping  Red Fescue | 6.0 | 30.0 |
| ELS | Wild bird seed (biennial) | *× Triticosecale* | Triticale (var. Logo) | 27.2 | 67.9 |
| ELS | Wild bird seed (biennial) | *Beta vulgaris* | Beet | 0.8 | 2.0 |
| ELS | Wild bird seed (biennial) | *Brassica oleracea Acephala Group* | Kale (var. Thousand head) | 6.0 | 15.0 |
| ELS | Wild bird seed (biennial) | *Chenopodium quinoa* | Quinoa | 6.0 | 15.0 |
| ELS | Wild bird seed (biennial) | *Cichorium intybus* | Chicory (var. Puna) | 0.04 | 0.1 |
| ELSX | Tall grass with flowers | *Dactylis glomerata* | Cock's-foot | 5.7 | 28.5 |
| ELSX | Tall grass with flowers | *Festuca arundinacea* | Tall Fescue | 2.9 | 14.3 |
| ELSX | Tall grass with flowers | *Festuca pratensis* | Meadow Fescue | 4.8 | 23.8 |
| ELSX | Tall grass with flowers | *Festuca rubra* | Red Fescue | 3.8 | 19.0 |
| ELSX | Tall grass with flowers | *Phleum pratense* | Timothy | 1.9 | 9.5 |
| ELSX | Tall grass with flowers | *Achillea millefolium* | Yarrow | 0.1 | 0.5 |
| ELSX | Tall grass with flowers | *Centaurea nigra* | Common Knapweed | 0.3 | 1.5 |
| ELSX | Tall grass with flowers | *Daucus carota* | Wild Carrot | 0.3 | 1.3 |
| ELSX | Tall grass with flowers | *Dipsacus fullonum* | Wild Teasel | 0.2 | 0.8 |
| ELSX | Tall grass with flowers | *Lotus corniculatus* | Common Bird's-foot-trefoil | 0.1 | 0.5 |
| ELSX | Tall grass with flowers | *Vicia cracca* | Tufted Vetch | 0.1 | 0.5 |
| ELSX | Pollen & nectar | *Lotus cornculatus* | Birdsfoot Trefoil  (var. Sans Gabriel) | 3.0 | 20.0 |
| ELSX | Pollen & nectar | *Onobrychis viciifolia* | Sainfoin | 6.0 | 40.0 |
| ELSX | Pollen & nectar | *Trifolium hybridum* | Alsike Clover (var. Aurora) | 2.3 | 15.0 |
| ELSX | Pollen & nectar | *Trifolium pratense* | Red Clover | 3.8 | 25.0 |
| ELSX | Wild bird seed (Bumblebird) | *× Triticosecale* | Triticale (var. Logo) | 0.6 | 3.0 |
| ELSX | Wild bird seed (Bumblebird) | *Borago officinalis* | Borage | 1.4 | 7.0 |
| ELSX | Wild bird seed (Bumblebird) | *Chenopodium quinoa* | Quinoa | 8.0 | 40.0 |
| ELSX | Wild bird seed (Bumblebird) | *Echinochloa esculenta* | White Millet | 1.1 | 5.6 |
| ELSX | Wild bird seed (Bumblebird) | *Helianthus annuus* | Semi-Dwarf Sunflower | 2.0 | 10.0 |
| ELSX | Wild bird seed (Bumblebird) | *Melilotus officinalis* | Sweet Clover | 3.0 | 15.0 |
| ELSX | Wild bird seed (Bumblebird) | *Raphanus sativus* | Fodder radish (var. Apoll) | 2.0 | 10.0 |
| ELSX | Wild bird seed (annual) | *× Triticosecale* | Triticale (var. Logo) | 16.0 | 40.0 |
| ELSX | Wild bird seed (annual) | *Chenopodium quinoa* | Quinoa | 4.0 | 10.0 |
| ELSX | Wild bird seed (annual) | *Echinochloa frumentacea* | White Millet | 8.0 | 20.0 |
| ELSX | Wild bird seed (annual) | *Fagopyrum esculentum* | Buckwheat | 8.0 | 20.0 |
| ELSX | Wild bird seed (annual) | *Raphanus sativus* | Fodder radish (var. Apoll) | 4.0 | 10.0 |
| ELSX | Wild bird seed (biennial) | *× Triticosecale* | Triticale (var. Logo) | 27.2 | 67.9 |
| ELSX | Wild bird seed (biennial) | *Beta vulgaris* | Beet | 0.8 | 2.0 |
| ELSX | Wild bird seed (biennial) | *Brassica oleracea Acephala Group* | Kale (var. Thousand head) | 6.0 | 15.0 |
| ELSX | Wild bird seed (biennial) | *Chenopodium quinoa* | Quinoa | 6.0 | 15.0 |
| ELSX | Wild bird seed (biennial) | *Cichorium intybus* | Chicory (var. Puna) | 0.04 | 0.1 |
| ELSX | Wildflower | *Agrostis capillaris* | Common Bent | 1.1 | 5.6 |
| ELSX | Wildflower | *Cynosurus cristatus* | Crested Dogstail | 7.9 | 39.4 |
| ELSX | Wildflower | *Festuca rubra ssp commutata* | Chewings Fescue | 3.4 | 16.9 |
| ELSX | Wildflower | *Festuca rubra* | Slender Creeping  Red Fescue | 5.6 | 28.1 |
| ELSX | Wildflower | *Achillea millefolium* | Yarrow | 0.1 | 0.3 |
| ELSX | Wildflower | *Centaurea nigra* | Common Knapweed | 0.2 | 0.8 |
| ELSX | Wildflower | *Clinopodium vulgare* | Wild Basil | 0.1 | 0.3 |
| ELSX | Wildflower | *Daucus carota* | Wild Carrot | 0.1 | 0.3 |
| ELSX | Wildflower | *Filipendula ulmaria* | Meadowsweet | 0.1 | 0.3 |
| ELSX | Wildflower | *Galium mollugo* | Hedge Bedstraw | 0.0 | 0.2 |
| ELSX | Wildflower | *Galium verum* | Lady’s Bedstraw | 0.1 | 0.5 |
| ELSX | Wildflower | *Knautia arvensis* | Field Scabious | 0.1 | 0.5 |
| ELSX | Wildflower | *Leontodon hispidus* | Rough Hawkbit | 0.0 | 0.2 |
| ELSX | Wildflower | *Leucanthemum vulgare* | Oxeye Daisy | 0.1 | 0.4 |
| ELSX | Wildflower | *Lotus corniculatus* | Birdsfoot Trefoil | 0.1 | 0.4 |
| ELSX | Wildflower | *Lychnis flos-cuculi* | Ragged Robin | 0.0 | 0.2 |
| ELSX | Wildflower | *Malva moschata* | Musk Mallow | 0.1 | 0.5 |
| ELSX | Wildflower | *Plantago media* | Hoary Plantain | 0.1 | 0.3 |
| ELSX | Wildflower | *Primula veris* | Cowslip | 0.1 | 0.5 |
| ELSX | Wildflower | *Prunella vulgaris* | Selfheal | 0.1 | 0.5 |
| ELSX | Wildflower | *Ranunculus acris* | Meadow Buttercup | 0.2 | 1.0 |
| ELSX | Wildflower | *Rumex acetosa* | Common Sorrel | 0.1 | 0.5 |
| ELSX | Wildflower | *Sanguisorba minor ssp minor* | Salad Burnet | 0.2 | 0.8 |
| ELSX | Wildflower | *Silene dioica* | Red Campion | 0.1 | 0.5 |
| ELSX | Wildflower | *Silene vulgaris* | Bladder Campion | 0.1 | 0.3 |
| ELSX | Wildflower | *Stachys officinalis* | Betony | 0.1 | 0.3 |
| ELSX | Wildflower | *Trifolium pratense* | Red Clover | 0.1 | 0.5 |
| ELSX | Wildflower | *Vicia cracca* | Tufted Vetch | 0.1 | 0.3 |

**Additional analysis of wheat and oilseed rape**

Fig S1: Yield of wheat (mean ±SE) as a ratio of regional yields averaged over all years (2006-11) for a) Cropped area and b) Whole field net of land removed for wildlife habitat creation.

Fig S2: Yield of oilseed rape (mean ±SE) as a ratio of regional yields averaged over all years (2006-11) for a) Cropped area and b) Whole field net of land removed for wildlife habitat creation.

**4) Additional analysis of nutritional value and profitability**

The differences between treatments for calculated energy produced and monetary value for a simple five year rotation are shown in Fig S3a,b. This should be balanced against the 3% and 8% of land removed from production to create wildlife habitats for the ELS and ELS Extra treatments respectively. The ELS treatment produced 0.6% more energy than the business as usual control, the ELS Extra treatments 2.2% less energy. In terms on monetary value, the ELS treatment was 0.7% higher than the control and ELS Extra treatment was 2.8% less profitable. In all cases the calculated confidence intervals (CI’s) of business as usual control, ELS & ELS Extra treatments overlapped (Table S2), meaning no significant differences between treatments (*p*>0.05).

Fig S3: Production (mean ±SE) of a) energy (MJ ha^-1^) and b) money (Gross Margins in Euro ha^-1^) derived from a standard 5 year crop rotation comprising: wheat-oilseed rape-wheat-beans-wheat in each of the three treatments.

Table S2: Calculated confidence intervals (CI’s) between treatments for energy produced and monetary value for a simple five year crop rotation. Upper means the +95% CI, lower means the –95% CI. The 95% CI is the CI value which is added or taken away from the mean.

| 1. **Energy (MJ ha^-1^)** | **Control** | **ELS** | **ELS Extra** |
| --- | --- | --- | --- |
| Mean | 157901933 | 158867521 | 154373719 |
| 95% CI | 9775629 | 8936289 | 9234420 |
| upper | 167677562 | 167803810 | 163608138 |
| lower | 148126304 | 149931233 | 145139299 |
|  |  |  |  |
| **b) Profit (Gross Margin Euros ha^-1^)** | **Control** | **ELS** | **ELS Extra** |
| Mean | 2414 | 2442 | 2346 |
| 95% CI | 149.1 | 139.1 | 143.5 |
| upper | 2563 | 2581 | 2489 |
| lower | 2264 | 2303 | 2202 |
